# Steroid-induced Diabetes Complicating Treatment of Epidermolysis Bullosa Acquisita: A Preventable Treatment Complication Stresses the Importance of Primary Care Follow-up

**DOI:** 10.7759/cureus.3608

**Published:** 2018-11-19

**Authors:** Victoria Byrd, Attila Nemeth

**Affiliations:** 1 Pediatrics, Case Western Reserve University School of Medicine, Cleveland, USA; 2 Internal Medicine, Louis Stokes Cleveland VA Medical Center/Case Western Reserve University School of Medicine, Cleveland, USA

**Keywords:** epidermolysis bullosa acquisita, epidermolysis bullosa acquisita, hyperglycemic hyperosmolar state, steroid-induced diabetes mellitus, hyperglycemic hyperosmolar state, patient follow up

## Abstract

Epidermolysis bullosa acquisita is a rare autoimmune bullous disease involving the skin and mucosa, most commonly treated with systemic corticosteroids. This case illustrates the importance of counseling patients on medication side effects and ensuring close physician follow-up during an extended course of steroids. A 46-year-old man presented to the emergency department with weakness, fatigue, dizziness and polyuria in the setting of eight weeks of prednisone therapy for a flare-up of his bullous disease. Labs were significant for a blood glucose of 786 mg/dL, negative urine ketones, a normal anion gap, and an acute kidney injury. Blood glucose improved to 413 mg/dL after initial treatment with fluid and insulin. The patient was admitted and acute kidney injury resolved. He remained hyperglycemic despite his adjusted prednisone taper and corrective scale insulin, so basal and scheduled, pre-prandial insulins were added. After discharge, he was bridged to steroid-sparing therapy (rituximab). Physicians should counsel patients with epidermolysis bullosa acquisita about the risks of steroid-induced diabetes mellitus and its associated complications including hyperglycemic hyperosmolar state and diabetic ketoacidosis. Primary care physicians should screen for hyperglycemia during therapy and consider alternative treatments when necessary.

## Introduction

Epidermolysis bullosa acquisita (EBA) is a rare acquired autoimmune subepidermal bullous disease of the skin and mucosa [1]. Systemic corticosteroids are the most commonly used medications to treat EBA, though good responses to alternative therapies have recently been reported [1-4]. Unfortunately, patients on long-term corticosteroid treatment are at risk for a myriad of adverse effects, including steroid-induced diabetes mellitus [5-7]. Here, we present the case of a patient with EBA who presented with hyperglycemic hyperosmolar state in the setting of a prolonged oral prednisone taper for an exacerbation of his disease.

## Case presentation

A 46-year-old man with past medical history significant for epidermolysis bullosa acquisita, prediabetes, and dyslipidemia presented to our emergency department (ED) with fatigue, polyuria, and weakness. The patient had seen his dermatologist eight weeks prior to presentation for a flare-up of epidermolysis bullosa acquisita, and was started on 60 mg of oral prednisone daily with a prolonged taper. His dose at the time of presentation was 30 mg. On the day of presentation, he reported two weeks of increased urinary frequency, dry mouth, diffuse muscle cramps, three days of weakness and fatigue, and one day of dizziness. He had been treated with prednisone several times since his diagnosis at age 14, but had never experienced these symptoms. A hemoglobin A1C obtained five months prior to admission was 6.4%, but the patient was not aware of a prediabetes diagnosis. He admitted to drinking sugary drinks regularly.

**Figure 1:**
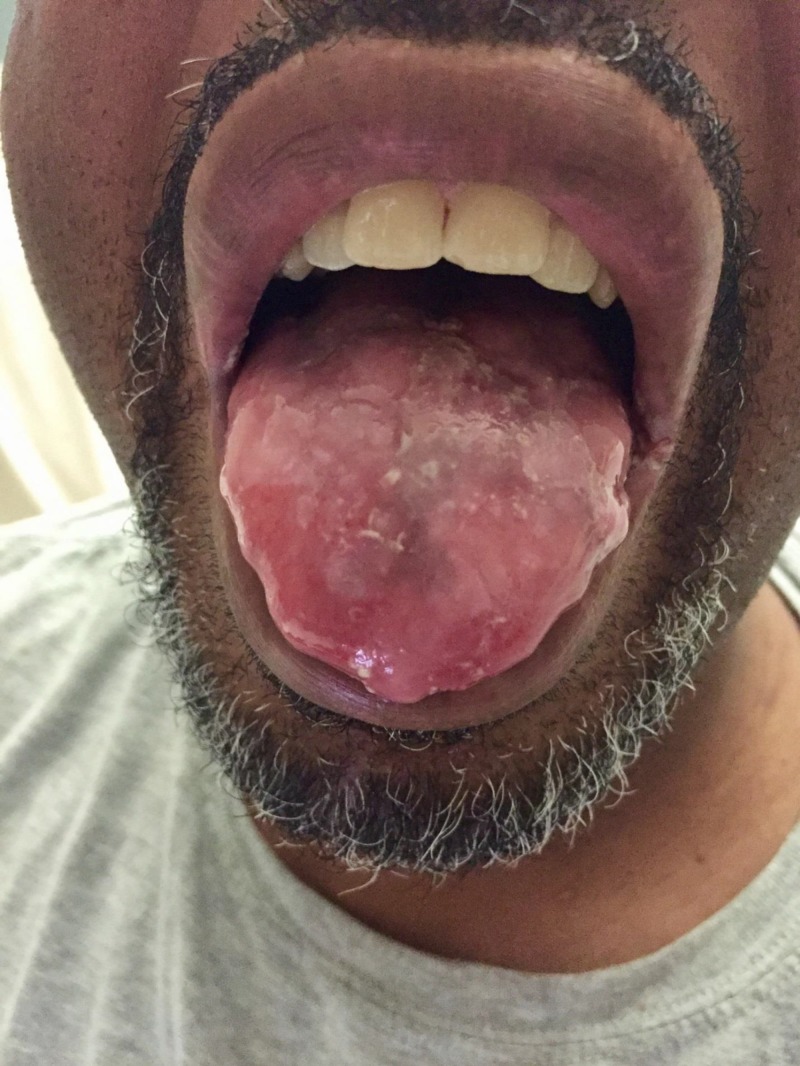
Tongue Bullae.

On examination, vitals revealed a temperature 98.1°F, pulse 96 beats per minute, respiratory rate 18 breaths per minute, and blood pressure 142/91 mmHg. Dry mucous membranes were present. Bullae were noted on the tongue, soft palate, and dorsal hands and elbows bilaterally. Figure 1 demonstrated the tongue bullae. Otherwise, physical exam was unremarkable.

Labs revealed blood glucose of 786 mg/dL and negative urine ketones. Anion gap was normal, so this presentation was consistent with hyperglycemic hyperosmolar state. The patient was also found to have an acute kidney injury with a serum creatinine of 2.8 mg/dL, up from his baseline of 1.2 mg/dL. This was suspected to be prerenal in the setting of dehydration, which was supported by a fractional excretion of sodium of 0.8%. The patient’s glucose decreased to 413 mg/dL with a one liter normal saline bolus and 8 units of intravenous regular insulin in the ED, and he was admitted to the medicine service for further management of hyperglycemia and acute kidney injury. Intravenous fluids were continued, and his creatinine gradually normalized. Dermatology was consulted and recommended an adjusted prednisone taper with an immediate change from 30 to 20 mg daily. On admission, the patient was initially started on only a sliding scale insulin regimen by the night float resident physician, but had multiple subsequent blood glucose readings in the 300-400 mg/dL range. The next morning when the patient was staffed with an attending physician glargine insulin was added. A repeat hemoglobin A1C was 11.9%. Endocrinology was consulted. The patient ultimately required 30 units of glargine insulin and 10 units of aspart insulin with meals. He was discharged on this regimen after receiving diabetic supplies and insulin administration education.

When the patient followed up one week after discharge, his average blood glucose reading was 300 mg/dL despite taking insulins as prescribed. Aspart insulin was increased to 12 units, with a good response. The patient’s outpatient dermatologist then noted that his epidermolysis bullosa acquisita was active despite adherence to the adjusted prednisone taper, and he was bridged to a steroid-sparing therapy (rituximab).

## Discussion

Patients are placed on steroids for treatment of a myriad of conditions, ranging from pulmonary to rheumatologic to dermatologic conditions. Steroids are expected to cause hyperglycemia. The hyperglycemia is tolerated in the short term while the patient is on the steroids. For example, if a patient is on a five-day steroid taper for a gout flare, monitoring blood glucose levels in a nondiabetic or pre-diabetic patient is not usually performed. However, if a patient requires steroids for a prolonged period of time, usually on the order of weeks to months, or if they are on prolonged taper over several months, it is prudent to monitor their blood glucose.

Patients at risk should be screened for hyperglycemia during the first 1-2 days of steroid therapy, when most cases of SIDM develop [5]. Steroids cause primarily post-prandial hyperglycemia, so post-prandial blood glucose readings are more sensitive than fasting glucose tests [7, 8]. SIDM is best diagnosed by an oral glucose tolerance test [7, 9]. Insulin is the treatment of choice in most cases. A short-acting prandial insulin administration scheme may be adequate, but patients with persistent hyperglycemia >140 mg/dL require the addition of a basal long-acting insulin [5]. The prognosis for SIDM is good. As patients are tapered to lower steroid doses, their endocrine function generally returns to baseline and insulin requirements decrease [5]. Long-term low-dose glucocorticoid use has been linked to increased cardiovascular mortality, possibly due to the microvascular complications of hyperglycemia [7]. The timely identification of SIDM, when not preventable, is therefore crucial so that cardiovascular risk factors can be optimized and good glycemic control can be achieved early in the patient’s course.

There are many risk factors for developing SIDM. The longer duration and higher dose of continuous glucocorticoid therapy are risk factors for SIDM [5]. Other risk factors include increased age, body mass index (BMI), and hemoglobin A1c [5]. Prediabetes is also a proposed risk factor for SIDM [6]. It is important to identify patients undergoing steroid treatment who are at risk for SIDM, as diabetes may go undetected in a previously normal or prediabetic individual.

SIDM was not an unpredictable side effect of long-term steroid treatment in an individual with prediabetes and EBA, and his hospitalization could likely have been avoided if the patient had received close follow-up with his primary care physician after being started on high-dose steroid therapy by his outpatient dermatologist. This hospital admission may have represented a communication breakdown on two levels. First, the patient in this case was unaware of his prediabetes diagnosis and the associated increased risk of SIDM. This patient should have been counseled about the risks, along with the signs and symptoms, of developing SIDM while treating EBA with steroids. Secondly, there was a communication breakdown between the patient’s primary care physician and his dermatologist, which demonstrates the importance of inter-specialty communication and coordination of care.

## Conclusions

This case demonstrates a scenario in which steroid-induced diabetes associated with the treatment of epidermolysis bullosa acquisita presented as hyperglycemic hyperosmolar state, due to a patient being lost to follow up. This was not an unpredictable side effect of long-term steroid treatment in a patient with prediabetes. However, the important learning point is that this admission may have been avoided if the patient had received close follow-up with his primary care physician after being started on high-dose steroid therapy by his outpatient dermatologist. The second learning point is that communication between primary care and specialists regarding a patient treatment's plan is vital.
